# Fitness costs of individual and combined pyrethroid resistance mechanisms, *kdr* and CYP-mediated detoxification, in *Aedes aegypti*

**DOI:** 10.1371/journal.pntd.0009271

**Published:** 2021-03-24

**Authors:** Letícia B. Smith, Juan J. Silva, Connie Chen, Laura C. Harrington, Jeffrey G. Scott

**Affiliations:** Department of Entomology, Comstock Hall, Cornell University, Ithaca, New York, United States of America; Universita degli Studi di Pavia, ITALY

## Abstract

**Background:**

*Aedes aegypti* is an important vector of many human diseases and a serious threat to human health due to its wide geographic distribution and preference for human hosts. *A*. *aegypti* also has evolved widespread resistance to pyrethroids due to the extensive use of this insecticide class over the past decades. Mutations that cause insecticide resistance result in fitness costs in the absence of insecticides. The fitness costs of pyrethroid resistance mutations in *A*. *aegypti* are still poorly understood despite their implications for arbovirus transmission.

**Methodology/Principle findings:**

We evaluated fitness based both on allele-competition and by measuring specific fitness components (i.e. life table and mating competition) to determine the costs of the different resistance mechanisms individually and in combination. We used four congenic *A*. *aegypti* strains: Rockefeller (ROCK) is susceptible to insecticides; KDR:ROCK (KR) contains only *voltage-sensitive sodium channel* (*Vssc*) mutations S989P+V1016G (*kdr*); CYP:ROCK (CR) contains only CYP-mediated resistance; and CYP+KDR:ROCK (CKR) contains both CYP-mediated resistance and *kdr*. The *kdr* allele frequency decreased over nine generations in the allele-competition study regardless of the presence of CYP-mediated resistance. Specific fitness costs were variable by strain and component measured. CR and CKR had a lower net reproductive rate (R_0_) than ROCK or KR, and KR was not different than ROCK. There was no correlation between the level of permethrin resistance conferred by the different mechanisms and their fitness cost ratio. We also found that CKR males had a reduced mating success relative to ROCK males when attempting to mate with ROCK females.

**Conclusions/Significance:**

Both *kdr* and CYP-mediated resistance have a fitness cost affecting different physiological aspects of the mosquito. CYP-mediated resistance negatively affected adult longevity and mating competition, whereas the specific fitness costs of *kdr* remains elusive. Understanding fitness costs helps us determine whether and how quickly resistance will be lost after pesticide application has ceased.

## Author summary

Insecticides are still one of the most important tools, and often only tool, used to control disease vectors such as mosquitoes. The mosquito *Aedes aegypti* transmits several important pathogens impacting human health, including dengue and Zika viruses. Pyrethroid insecticides are commonly used to control *A*. *aegypti* in endemic areas, however, heavy use of insecticides has resulted in insecticide resistant mosquitoes. In *A*. *aegypti*, pyrethroid resistance evolved primarily through mutations in the pyrethroid target site (here referred to as *kdr*) and by increased detoxification by CYP enzymes. In this study, we sought to understand how these resistance modifications impact the mosquito fitness, since mutations often come with a biological cost. We found that both mechanisms have a cost and that they affect different aspects of mosquito physiology. CYP-mediated detoxification has a greater negative impact in the mosquito longevity and its ability to compete for mates. *Kdr* has a clear cost, but the specific function that is affected was neither reproduction nor longevity and therefore remains elusive. This study helps us understand how resistance influences mosquito life history traits and may help in the design of more targeted insecticide resistance management strategies.

## Introduction

Insecticide resistance mutations and their effect on organismal fitness may play an important role in the dynamics of the evolution of insecticide resistance [1]. Resistance alleles have a cost to the individuals carrying them when there is no insecticide present (in the absence of a compensatory mutation) [2–5]. The rate of resistance evolution in a population is directly related to the intensity of the selection pressure, allele frequency, dominance, and relative fitness of the resistance versus susceptible genotypes [6]. Fitness costs associated with insecticide resistance can manifest in numerous ways, including disadvantages in development, reproduction, and survival. However, costs associated with specific resistance mechanisms are largely unknown and how multiple fitness costs might interact within an organism is even more unclear. Understanding the fitness effects and evolution of resistance is critical in integrated resistance management practices to help determine whether and how quickly resistance will be lost under field conditions after pesticide application has ceased.

Effective management of insecticide resistance is critical in disease vectors, including the anthropophilic mosquito, *Aedes aegypti*, which is responsible for the spread of dengue, yellow fever, chikungunya, and Zika viruses worldwide. Its wide global distribution and its ability to thrive in urban environments makes it a serious threat to human health. Dengue virus is the most important pathogen transmitted by *A*. *aegypti*. An estimated 50 to 100 million dengue virus infections occur worldwide annually [7,8]. Yellow fever, like dengue, is a viral hemorrhagic fever that can be lethal. Despite the existence of a preventative vaccine, there are still about 200,000 cases and 30,000 deaths of yellow fever worldwide each year [9] and these values are on the rise due to a recent outbreak in Brazil [10]. Chikungunya fever is a re-emerging mosquito-borne disease caused by chikungunya virus [11,12]. Zika has generated a great deal of human health concern since it reached the Americas in 2014 because of its rapid spread and associations with microcephaly and Guillain-Barré syndrome [13–16].

Pyrethroid insecticides, such as permethrin, are widely used for mosquito control, including control of adult *A*. *aegypti*. Insecticides are still the primary means to control *A*. *aegypti* in dengue endemic areas, which has led to extensive use of pyrethroids in the past decades. As a consequence, pyrethroid resistant *A*. *aegypti* populations are now found worldwide [17,18].

The two major mechanisms of pyrethroid resistance in *A*. *aegypti* are detoxification by cytochrome P450 monooxygenases (CYPs) and mutations in the target site, *voltage-sensitive sodium channel* gene (*Vssc*) [19]. In resistant strains, the increased expression of *CYP* genes or non-synonymous mutations in CYP proteins can increase the metabolism of insecticides into less-toxic metabolites, while mutations in *Vssc* make the channel less sensitive to pyrethroid insecticides. A very well characterized pyrethroid resistant strain of *A*. *aegypti* is Singapore (SP) [19]. In SP, resistance is due to the overexpression of CYPs [20] and two *Vssc* mutations, S989P+V1016G (referred to as *knock down resistance* or *kdr*) [19,20].

Fitness costs can be evaluated either using a population cage study where the resistance levels or resistance allele frequencies of populations are tracked over time, or by measuring individual fitness components, such as survival, reproduction, and growth, in a life history study. Both methods have their advantages and disadvantages. In a population cage study, the overall presence and magnitude of a cost can be measured, but the specific component(s) responsible for the observed cost remains elusive. Allele-competition studies also have the advantage of being easily adapted to wild populations in a field study, provided resistance markers are known. In a life history study, the presence and magnitude of potential costs can be measured (e.g. as decreased fecundity). However, the fitness cost could be missed if the measurements taken do not encompass parameters related to the fitness cost. There is, therefore, a higher chance of documenting and defining fitness costs when both methods are used. Fitness is dependent on the environment, and under laboratory conditions the number of biotic and abiotic stressors are more limited than under field conditions, so choice of conditions must be considered carefully [2]. To reasonably associate fitness differences with resistance, fitness studies are optimally performed using isogenic (or congenic) strains [21]. This is also especially important in the case where a laboratory susceptible strain is used for the comparison, because such a strain will be adapted to laboratory conditions, and thus likely perform better than a more recently field collected strain.

Results from previous fitness studies on pyrethroid resistant insects are variable, but also variable are the mutations, mechanisms, and components measured in these studies. We know, however, that resistance usually confers a fitness cost at some level [5]. All ten studies that measured reversion of either pyrethroid resistance alleles or resistance levels found a decrease over time [2,3,22–29]. Similarly, four out of five studies that calculated a net reproductive rate (R_0_) or intrinsic rate of increase (r or r_m_) found a reduced reproductive output for pyrethroid resistant strains [27,30–33]. When measuring specific components, a general trend is less clear. In general, more studies found a cost in some reproductive (about 64%) and developmental (about 63%) component, but fewer saw a cost due to longevity (about 47%) or body size (about 43%), and none have reported a cost due to sex ratio [3,27,30–32,34–48]. Part of this variation is likely a result of the different mutations and mechanisms (and their combinations) being tested, since these are likely to confer different types of fitness costs. The shortage of fitness studies with known resistance mechanisms makes it difficult to draw general conclusions on the fitness costs of pyrethroid resistance and in particular, on the costs of the different resistance mechanism.

Interactions between CYP-mediated resistance and *kdr* can influence the rate at which pyrethroid resistance evolves and therefore, influence the level of resistance in a population. Few studies have looked at the interaction between different insecticide resistance mechanisms and how this affects resistance levels [49,50], and none have also looked at the effects of this interaction on fitness. Understanding this interaction would give us new insight into the evolution of these mechanisms.

The goal of this study was to understand the evolution of the two main mechanisms of resistance, *kdr* and CYP-mediated detoxification, in *A*. *aegypti* by looking at what fitness costs do they have individually and collectively in the absence of insecticides. Three congenic resistant strains of *A*. *aegypti* were used for comparisons: one with only the *Vssc* mutations 998P+1016G (KDR:ROCK or KR), one with only the CYP-mediated detoxification mechanism (CYP:ROCK or CR), and one containing both *kdr* plus CYP-mediated resistance (CYP+KDR:ROCK or CKR). The individual and combined contributions to insecticide resistance of these two *A*. *aegypti* resistance mechanisms, *kdr* and CYP-mediated detoxification, were assessed in previous studies [50,51]. Specifically, we addressed the following questions: What are the fitness costs of the individual resistance mechanisms? When both mechanisms are present, are the fitness costs additive, greater-than, or less-than additive? And what is the relationship between the resistance levels and fitness costs of the individual and combined resistance mechanisms? The fitness costs were evaluated both based on specific fitness components (i.e. adult and larval life tables, and mating competition) and by measuring overall fitness via an allele-competition study. The results from this study contribute to our understanding of the evolution of pyrethroid resistance by providing information on how these genes interact in regard to both their advantages and disadvantages for the organism. This also has implications for studies of fitness costs and for insecticide resistance management strategies because the genetic interactions between these two resistance mechanisms can affect the rate in which resistance will spread in a population.

## Materials and methods

### Mosquito strains

Four congenic strains of *A*. *aegypti* were used in this study: Rockefeller (ROCK), a well-studied insecticide-susceptible strain [52], and three permethrin resistant strains with different resistance mechanisms (originating from the Singapore (SP) strain) introgressed into the ROCK genetic background. KDR:ROCK (KR) has *Vssc* mutations S989P+V1016G (*kdr*) as the only mechanism of resistance [51], CYP:ROCK (CR) has only CYP-mediated resistance and no *kdr* [50] and CYP+KDR:ROCK (CKR) has *kdr* and CYP-mediated resistance [20]. At the start of the allele-competition study (see next section), the KR strain was found to contain 4.4% heterozygous individuals (S1 Table). An additional genotype selection for homozygous *kdr* individuals was done on 630 females and 180 males prior to the fitness component studies (see Fitness component studies section) to ensure the following generations of KR were homozygous for *kdr*. Both *kdr* mutations in KR occur in the same domain (Domain II) of *Vssc* and recombination (sites are ~310 bp apart) has not been observed [53,54].

### Allele competition experiments

**Fig 1:**
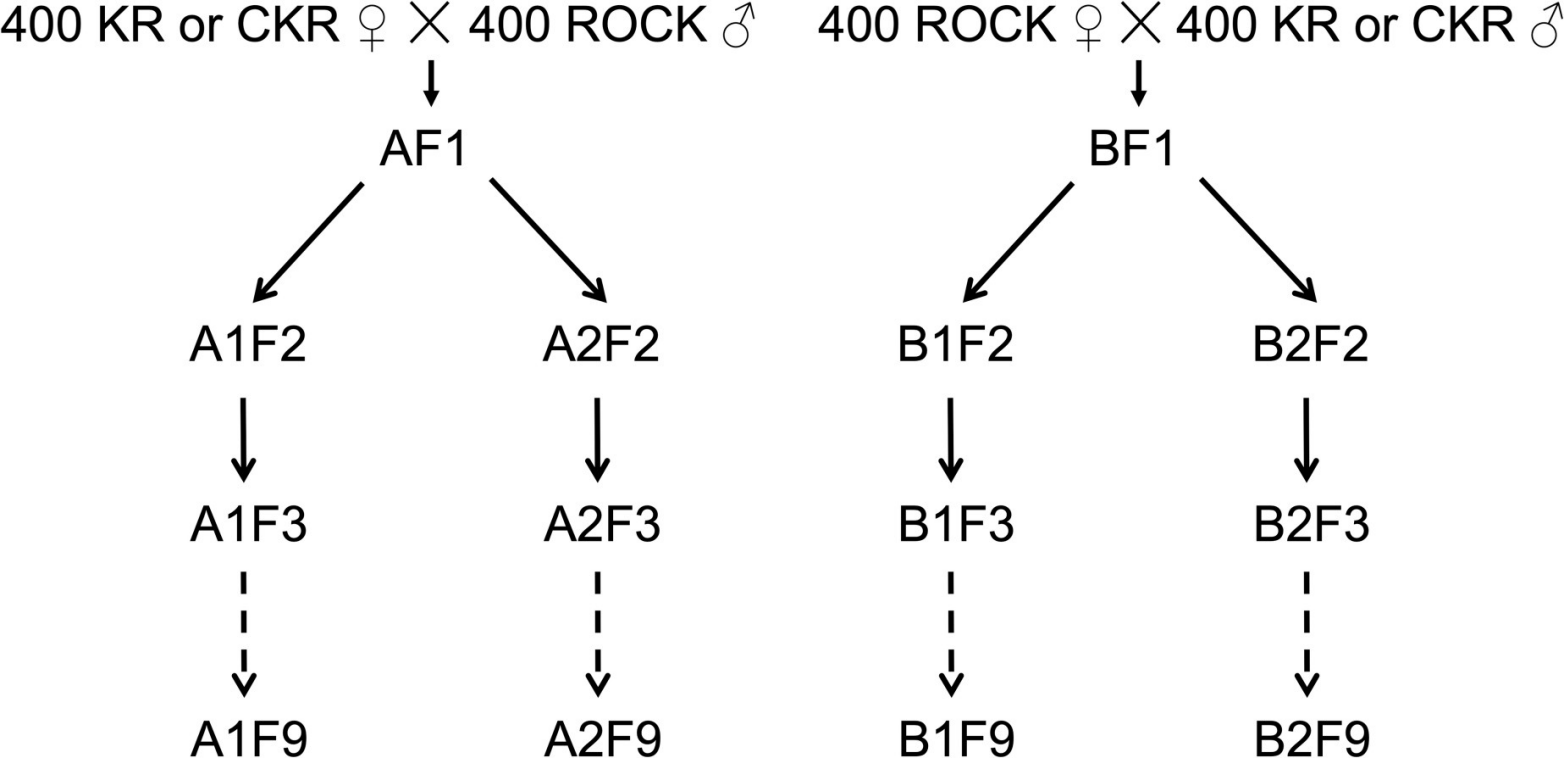
**Allele-competition reciprocal (A and B) crosses.** Two separate experiments were conducted: one crossing KR with ROCK and one crossing CKR with ROCK. Both experiments were maintained for nine generations.

Two independent allele competition lines were created to determine the fitness cost of *kdr* alone and in combination with CYP-mediated resistance: To look at *kdr* alone, KR and ROCK strains were crossed. To examine *kdr* in combination with CYP-mediated resistance, CKR and ROCK were crossed (Fig 1). Because the mutation causing CYP-mediated resistance is still unknown, we cannot track the CYP resistance genotype, and thus could not repeat this experiment with the CR strain. Reciprocal crosses A (KR or CKR females x ROCK males) and B (ROCK females x KR or CKR males) were set-up by releasing 400 virgin females and 400 virgin males of each parental strain in the appropriate cage and mosquitoes were left to mate *en masse* for 7 days. Crosses A and B were split into two replicate cages each (A1, A2, B1, B2) making a total of four cages per experiment. Mosquitoes were reared in a room with temperatures ranging from 25–30°C (median and average = 28), 3–37% (median = 14, average = 16) relative humidity, and a 14:10-h (light/dark) photoperiod. Due to physical limitations in rearing space the allele competition experiments with KR x ROCK and CKR x ROCK were carried out from April 2016-March 2017 and April 2017-January 2018, respectively. Females were blood fed using membrane covered water-jacketed glass feeders with cow blood obtained locally (Owasco Meat Co. Inc., Moravia, NY). Adults were maintained on 10% sucrose water in cages approximately 36 x 25 x 25 cm. Larvae were reared in 29.5 x 23 x 8.4 cm plastic containers (LOCK&LOCK Co., Ltd., Seocho-gu, Korea) with ~1.5 L distilled water and Hikari Cichlid Gold fish food pellets (Hikari, Hayward, CA, USA) (ground pellets for 1st instar and whole medium size pellets for 2nd to 4th instars). Food pellets were given daily, as needed depending on larval density (between approximately 600–1000) and instar. For each strain, two larval containers were maintained. Approximately 600–1000 pupae from the larval containers were picked to go into cages for each following generation. Additional emerging males and females were stored separately in 2 ml Eppendorf tubes at -80°C until they were genotyped.

Genomic DNA was extracted from the legs (~3–6 legs) of individual mosquitoes using an alkali extraction method as follows. Legs were placed in individual wells of a 96-well PCR plate (BioRad, Hercules, CA, USA) with approximately three 2.3-mm diameter zirconia/silica beads (BioSpec Products, Bartlesville, OK, USA) and 10 μL 0.2 M NaOH per tube. The samples were pulverized for 1–2 min on a vortex at maximum speed and then incubated for 10 min at 70°C. Ten microliters of a neutralization buffer (360 mM Tris-HCl, pH 7.5 and 10 mM EDTA) and 80 μL dH_2_0 were then added to each well.

For generations F_1_, F_3_, F_5_, F_7_, and F_9_, the *kdr* genotypes were determined for a minimum of 85 mosquitoes from each cage (equal numbers of males and females) using allele-specific polymerase chain reaction (ASPCR). Forward and reverse primers (Table 1) were made for both the *kdr* (resistant) and susceptible genotypes as described previously [55]. Resistant (R) and susceptible (S) primers were used on each sample with the following thermocycler conditions: 94°C for 3 min, 35 x (94°C for 30 sec, 62°C for 30 sec, 72°C for 1 min) and 72°C (7 min). Each ASPCR reaction was evaluated on a 1% agarose gel and was scored as homozygous susceptible (ASPCR band only with S primers), homozygous for *kdr* (ASPCR band only with R primers) or heterozygous (ASPCR band with both R and S primers). Controls (2 x each of ROCK, SP, and F_1_) were run on every 96-well plate.

**Table 1:** *Vssc* primers used for genotype selection and sequencing.

Primer name	Sequence (5’ to 3’)	Purpose
AaSCF20	GACAATGTGGATCGCTTCCC	Domain II PCR amplification (Forward)
AaSCR21	GCAATCTGGCTTGTTAACTTG	Domain II PCR amplification (Reverse)
AaSCR22	TTCACGAACTTGAGCGCGTTG	Domain II sequencing
Ser 1F	GCG GCG AGT GGA TCG AAT	Allele-specific susceptible genotype (Forward)
Val 1R	GCA AGG CTA AGA AAA GGT TAA GTA	Allele-specific susceptible genotype (Reverse)
Pro 1F	GCG GCG AGT GGA TCG AAC	Allele-specific resistant genotype (Forward)
Gly 1R	GCA AGG CTA AGA AAA GGT TAA GTC	Allele-specific resistant genotype (Reverse)

### Allele competition data analysis

Deviation from Hardy-Weinberg equilibrium (HWE) between generations was assessed using a chi-square test (χ^2^), with the genotype frequency of the previous generation used as the expected genotype frequencies. The level of significance for statistical analyses was p < 0.05 unless otherwise indicated. Simulations were used to assess the likelihood of the observed allele frequency changes to result from genetic drift alone. Simulations were done with R software [56] and scripts applied as in a previous study [2]. The model assumed a diploid and panmictic population with a fixed size of 800 individuals and the initial R allele frequency for each generation was defined as the observed frequency in the previous interval. Simulations were repeated 10,000 times per generation and p-values were estimated as the number of simulations in which ending allele frequency was equal or more extreme than the initial value of the interval divided by the total number of simulations. The null hypothesis assumed all fluctuations in allele frequency could be explained by genetic drift.

Differences between genotype and allele frequencies across generations were tested using a linear mixed model (using the lme4 package (v 1.2–21) in R 3.5.3, based on [57]) and checked for significance from F-values generated from ANOVA (using the lmerTest package (v 3.1–0) in R [58]). Pairwise comparisons between genotype and allele frequencies were done using Tukey’s method (using the emmeans package (v 1.3.3) in R (https://github.com/rvlenth/emmeans)) to determine significant differences (p < 0.05) across generations. A linear regression analysis was used to determine if the change in allele frequency was significantly associated with generation time. In addition to the regression analysis, a Fisher’s combined probability test of the HWE p-values was also used to determine if allele frequency changes across all the generations are in Hardy-Weinberg equilibrium, as a proxy to determine if the changes are due to selection. This method considers multiple tests with the same hypothesis and assesses the probability that all hypotheses are true in order to obtain a global p-value [59,60]. This test was also used to obtain a global p-value for genetic drift effect.

### Fitness component studies

ROCK, KR, CR, and CKR mosquitoes were reared in a chamber at 70–80% RH and 27°C ± 1°C with a photoperiod of 14:10-h (light/dark). To ensure synchronized hatching, eggs were first soaked in dH_2_0 for 30 min, followed by 30 min in a vacuum. First instar larvae were then separated for use in either the female life table experiment (200 larvae per 27.5 x 21.5 x 7.5 cm container with ~ 2L dH_2_O) or the larval life table experiment (single larva per well of a six-well plate–see method below).

For the female life table experiment, approximately 200 pupae were transferred to eclosion cups inside 35 x 25 x 25 cm cages with access to 10% sucrose *ad libitum* for three days post-eclosion and to allow for mating. Three days post-eclosion, females were blood fed from a human volunteer (L.B.S.). For each strain, 50 blood-fed females were transferred to individual 0.5 L paper soup cartons (Choice, Clark Associates Companies, Lancaster, PA) with modified tulle lids where they stayed for the duration of the experiment (S1 Fig). Each carton also contained a cup (clear 30 ml, DART Container Corp., Mason, MI) filled with dH_2_0 and lined with strips of white paper towel (Pacific Blue, Georgia-Pacific Corp., Atlanta, GA) for egg laying and as a source of water for hydration. The 200 cartons were arranged randomly at different heights to ensure uniform exposure to any variations in temperature, humidity, and light inside the rearing chamber. A blood meal was offered every three days from a human volunteer (L.B.S.) and whether or not a female blood-fed was tracked for the duration of the experiment. Mosquitoes were given approximately 5 min to feed. Females that did not feed to completion or at all were given a second chance to feed (5 min), after which, if they did not feed, they were counted as not feeding for that day. To measure fecundity, cups were checked daily for eggs and replaced with fresh filter paper whenever eggs were laid or replenished with water as needed. Eggs were counted daily under a magnifying glass. Adult mortality was recorded daily. Once all females of a single strain died, the experiment was ended. To estimate the body size, wings were removed (at the axillary incision) from each mosquito after they died and placed on a piece of double-sided tape pressed between two microscope slides (75x25x1 mm, VWR International, LLC. Radnor, PA) (S2 Fig). The distance from the axillary incision to the apical margin (excluding the fringe of scale) was measured as described previously [61] on an Olympus SZX9 microscope (Olympus Corp., Shinjuku, Tokyo, Japan) equipped with an Olympus DP22 camera using imaging software (Olympus cellSens Standard version 1.17). The entire experiment was repeated three times using different cohorts of mosquitoes.

For the larval life table experiment, 60 first instar larvae (L1) were placed in individual wells of 6-well plates with approximately 13 ml dH_2_O each (total of 10 plates per strain). Approximately 4–5 mg of Cichlid Gold fish food pellets was delivered as a food slurry to each well to sustain the larvae throughout its entire development. Ecdysis (1–4 instars), pupation, adult emergence/sex, and mortality were recorded every 24 h to determine the mean development time for each stage and the sex ratio. The experiment was repeated four times with different mosquito cohorts.

To determine the viability of the eggs (i.e. fertility), mosquitoes were hatched using the vacuum hatch method as described above and 200 larvae were transferred to larval containers containing ~ 2L of dH_2_O. Pupae were transferred into test tubes until adult eclosion. Fifty females and fifty males (of each strain) were released into a 35 x 25 x 25 cm cage supplied with 10% sucrose water for *en masse* mating. Three days after eclosion, mosquitoes were blood fed from a human volunteer (L.B.S.). An egg-laying cup lined with white paper towel was placed in each cage 3 days after the blood meal and removed two days later. Water was kept in the cup for an additional day to allow for proper embryogenesis and then removed to allow the paper to dry for three days. Random pieces of the egg paper were cut for egg hatching. Eggs were hatched using the vacuum method described above plus an additional 24 h for hatching. Larvae were counted after the vacuum hatch and 24 h later. The number of eggs used for the egg viability experiment was counted using an Olympus SZX9 microscope equipped with an Olympus DP22 camera using imaging software (Olympus cellSens Standard version 1.17). The egg viability was calculated as the total number of larvae from the total number of eggs. This experiment was repeated eight times with different mosquito cohorts.

A mating competition experiment was done to determine if the *kdr* and CYP-mediated resistance has an impact on mating success of male mosquitoes. This experiment was done with the resistant CKR and KR strains (because their resistant genotype can be tracked) and the susceptible ROCK strains. Larvae were hatched and reared as described above. Pupae were transferred into test tubes until adult eclosion to ensure they were virgin prior to release into 35 x 25 x 25 cm cages. Adults were released into cages supplied with 10% sucrose water and allowed to mate *en masse* for two days. All cages included equal numbers (25 each) of ROCK and resistant males (CKR or KR), and 25 either CKR, KR or ROCK females. Females were allowed to blood feed using membrane covered water-jacketed glass feeders with cow blood obtained locally. Females that successfully blood-fed were placed individually into Drosophila vials (Narrow polypropylene vials, VWR International, LLC. Radnor, PA) lined with white paper towel for egg-laying, filled approximately 2 cm with dH_*2*_O, and closed with a cotton stopper. After three days, the eggs were allowed to dry inside the vials for two days and then hatched by adding dH_2_O to the vials once more. The offspring of individual females were genotyped via ASPCR in the same method as described in the allele competition experiment section to determine if the male parent was resistant (CKR or KR) or susceptible (ROCK). Two offspring from each female parent were genotyped in order to confirm cross results, since *A*. *aegypti* females are known to mate only once. This experiment was repeated fourteen times for CKR versus ROCK and twelve times for KR versus ROCK with different cohorts of mosquitoes.

### Population growth and life table calculations

The population growth components of the different strains were calculated based on the methods described in [62] from the survival, fecundity, egg viability, and sex ratio data obtained in the fitness component studies described above. The net reproductive rate (R_0_) was calculated with the equation R_0_ = ∑l_x_m_x_, where l_x_ = fraction of females alive at age x, and m_x_ = number of daughters born to surviving females at age x. More precisely, we used the life table data in the R0 formula as follows: ∑ {proportion of females that survived * [((# eggs laid * egg viability)/ number of females alive) * sex ratio]}. The R_0_ provides a value for how many times a population will multiply in each generation assuming no predation or resource limitation. The intrinsic rate of increase (r) was calculated using the equation ∑e^(-rx)^l_x_m_x_ = 1, where l_x_, m_x_, and x are the same values as described above. The intrinsic rate of increase gives us a value for the growth potential of a population. When r = 0, the population isn’t increasing or decreasing, if r > 0, the population is increasing, and if r < 0, the population is decreasing. The mean length of a generation (T) was calculated using the equation T = ln(R_0_)/r, where R_0_ and r are defined above. Population growth components were calculated for each replicate separately and a final value given as the average for all three replicates per strain. To evaluate the fitness cost of the different resistance mechanisms in relation to the fitness advantage (i.e. permethrin resistance level conferred by each mechanism), a fitness cost ratio was calculated using the following equation: 1 - (Final R_0_ of resistant strain/ Final R_0_ of ROCK) where the final R_0_ was the average final R_0_ value of all three replicates for each strain. To determine if the fitness cost effects of having both mechanisms combined in the same strain have a greater-than, less-than, or additive effect, a two-way ANOVA with an interaction term for additivity was used with each mechanism, CYP-mediated resistance and *kdr*, as a factor.

### Fitness component data analysis

Differences between strains for the life table experiments, mating competition, and growth components were tested using a linear mixed model (using the lme4 package (v 1.2–21) in R 3.5.3, based on Bates et al. 2015 [57]) and checked for significance from F-values generated from ANOVA (using the lmerTest package (v 3.1–0) in R 3.5.3 [58]). Pairwise comparisons between strains were done using the Tukey’s method (using the emmeans package (v 1.3.3) in R 3.5.3 (https://github.com/rvlenth/emmeans)) to determine statistical differences (p < 0.05) across strains.

## Results

### Allele competition experiment

**Fig 2:**
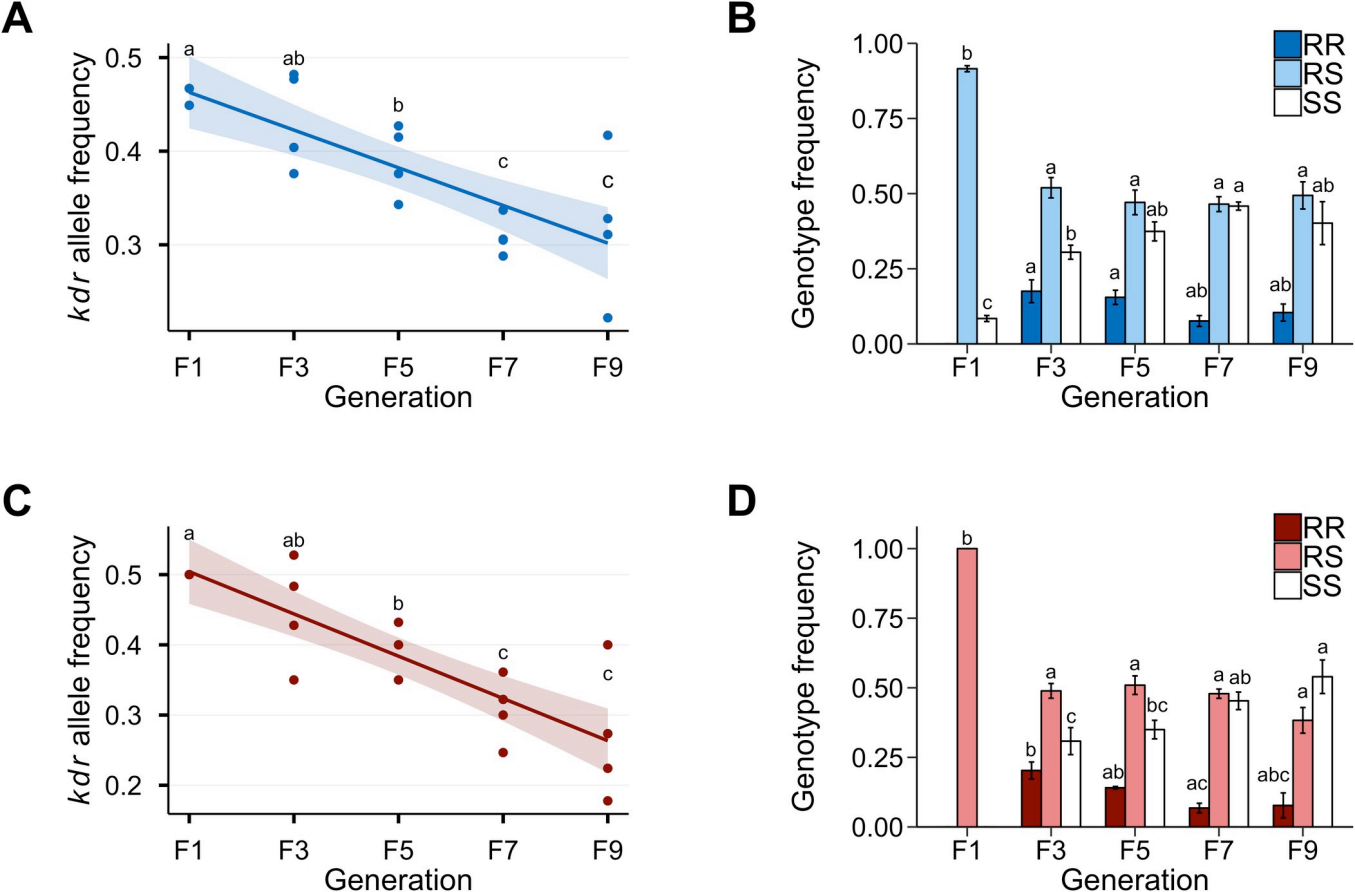
**Allele-competition results for the KR (A and B) and CKR (C and D) experiments.** Frequency of the *kdr* allele was significantly reduced from generation F_1_ to F_9_ for both KR (A) and CKR (C). The SS genotype (homozygous susceptible, white bars) increased significantly from generation F_3_ to F_7_ for both KR (B) and CKR (D), but was not significantly different from F_3_ to F_9_ for the KR experiment (B). The RR genotype changed significantly from F_3_ to F_7_ in the CKR experiment (B), but there was no significant difference in the KR experiment (D). Frequency of heterozygotes (RS) did not change between generations. Different letters represent significant differences (p < 0.05) of the susceptible and *kdr* alleles (A and C) or the SS, RS, and RR genotypes (B and D) across generations only. Shaded area around the line represent 95% CI (A and C) and bars represent the standard error (B and D).

In the absence of insecticide pressure, there was a fitness cost associated with *kdr* (*Vssc* mutations S989P+V1016G), both in the presence (Fig 2C and 2D) and absence (Fig 2A and 2B) of the CYP-mediated resistance. There was no significant difference between the reciprocal crosses A and B or between male and female samples, therefore the results were pooled to provide an overall change across generations. The frequency of the *kdr* allele decreased from an average of 0.46 to 0.32 (in KR, F_1,18_ = 29.12, p = 0.0000397, R^2^ = 0.596) and 0.50 to 0.27 (in CKR, F_1,18_ = 45.57, p = 0.00000251, R^2^ = 0.701) between the F_1_ and F_9_ generations (Fig 2A–2C, and Tables 2 and 3). The susceptible genotype (SS) increased from 0.31 at F_3_ for both KR and CKR to 0.46 and 0.54 at F_9_ for the KR and CKR experiments, respectively (Fig 2B–2D, Tables 2 and 3). This suggests that under these conditions and using these metrics, there is a fitness advantage to being homozygous susceptible (SS).

**Table 2:** Genotype and allele frequencies for each replicate of the KR x ROCK experiment and their associated Hardy-Weinberg (HWE) and genetic drift p-values.

Gen.	Sample size	Observed genotype frequency (expected^#^)			Allele frequency		HWE (p-value)	Genetic drift (p-value)
		SS	RS	RR	S	R		
AF_1_	90	0.07	0.93	0.00	0.53	0.47	--	--
BF_1_	88	0.10	0.90	0.00	0.55	0.45	--	--
A1F_3_	89	0.37 (0.39)	0.51 (0.47)	0.12 (0.14)	0.62	0.38	0.015*	0.000
A1F_5_	89	0.31 (0.33)	0.52 (0.49)	0.17 (0.18)	0.57	0.43	0.142	0.002
A1F_7_	85	0.45 (0.51)	0.53 (0.41)	0.02 (0.08)	0.71	0.29	0.000*	0.000
A1F_9_	178	0.60 (0.61)	0.37 (0.34)	0.04 (0.05)	0.78	0.22	0.006*	0.000
A2F_3_	89	0.29 (0.36)	0.61 (0.48)	0.10 (0.16)	0.60	0.40	0.005*	0.000
A2F_5_	88	0.33 (0.34)	0.51 (0.49)	0.16 (0.17)	0.59	0.42	0.564	0.273^
A2F_7_	87	0.47 (0.50)	0.45 (0.42)	0.08 (0.09)	0.70	0.30	0.003*	0.000
A2F_9_	180	0.30 (0.34)	0.57 (0.49)	0.13 (0.17)	0.58	0.42	0.000*	0.000
B1F_3_	88	0.26 (0.28)	0.52 (0.49)	0.22 (0.23)	0.52	0.48	0.378	0.047
B1F_5_	89	0.50 (0.39)	0.35 (0.47)	0.20 (0.14)	0.62	0.38	0.000*	0.000
B1F_7_	89	0.43 (0.44)	0.47 (0.45)	0.10 (0.11)	0.66	0.34	0.244	0.011
B1F_9_	90	0.50 (0.48)	0.40 (0.43)	0.11 (0.10)	0.69	0.31	0.330	0.059^
B2F_3_	88	0.30 (0.27)	0.44 (0.50)	0.26 (0.23)	0.52	0.48	0.152	0.023
B2F_5_	89	0.41 (0.44)	0.50 (0.44)	0.09 (0.12)	0.66	0.34	0.000*	0.000
B2F_7_	93	0.47 (0.45)	0.40 (0.44)	0.13 (0.11)	0.67	0.33	0.391	0.189^
B2F_9_	90	0.42 (0.45)	0.50 (0.44)	0.08 (0.11)	0.67	0.33	0.197	0.480^
Global χ^2^ (df = 32) =							143.28	198.07
Global p-value =							0.000*	0.000

# Expected genotype frequency values estimated from same generation

* Values out of HWE

^ Unable to reject null hypothesis of allele frequency changes due to genetic drift

**Table 3:** Genotype and allele frequencies for each replicate of the CKR x ROCK experiment and their associated Hardy-Weinberg (HWE) and genetic drift p-values.

Gen.	Sample size	Genotype frequency observed (expected^#^)			Allele frequency		HWE (p-value)	Genetic drift (p-value)
		SS	RS	RR	S	R		
AF_1_	90	0.00	1.00	0.00	0.50	0.50	--	--
BF_1_	90	0.00	1.00	0.00	0.50	0.50	--	--
A1F_3_	90	0.33 (0.33)	0.48 (0.49)	0.19 (0.19)	0.57	0.43	0.047*	0.000
A1F_5_	90	0.33 (0.36)	0.53 (0.48)	0.13 (0.16)	0.60	0.40	0.184	0.057^
A1F_7_	90	0.40 (0.46)	0.51 (0.44)	0.07 (0.11)	0.68	0.32	0.014*	0.000
A1F_9_	90	0.41 (0.37)	0.38 (0.47)	0.21 (0.17)	0.60	0.40	0.001*	0.000
A2F_3_	90	0.19 (0.22)	0.57 (0.50)	0.24 (0.28)	0.47	0.53	0.142	0.059^
A2F_5_	90	0.29 (0.32)	0.56 (0.49)	0.15 (0.19)	0.57	0.43	0.116	0.000
A2F_7_	90	0.39 (0.41)	0.50 (0.46)	0.11 (0.13)	0.64	0.36	0.046*	0.000
A2F_9_	89	0.47 (0.53)	0.51 (0.40)	0.02 (0.08)	0.73	0.27	0.002*	0.000
B1F_3_	90	0.29 (0.27)	0.46 (0.50)	0.26 (0.24)	0.52	0.48	0.340	0.164^
B1F_5_	90	0.33 (0.36)	0.53 (0.48)	0.13 (0.160	0.60	0.40	0.018*	0.000
B1F_7_	90	0.47 (0.49)	0.47 (0.42)	0.07 (0.09)	0.70	0.30	0.020*	0.000
B1F_9_	87	0.60 (0.60)	0.36 (0.35)	0.05 (0.05)	0.78	0.22	0.029*	0.000
B2F_3_	90	0.42 (0.42)	0.46 (0.45)	0.12 (0.12)	0.65	0.35	0.000*	0.000
B2F_5_	90	0.44 (0.42)	0.41 (0.45)	0.14 (0.12)	0.65	0.35	0.378	0.506^
B2F_7_	90	0.54 (0.57)	0.44 (0.36)	0.03 (0.07)	0.75	0.25	0.002*	0.000
B2F_9_	90	0.68 (0.68)	0.29 (0.29)	0.03 (0.03)	0.82	0.18	0.035*	0.000
Global χ^2^ (df = 32) =							125.46	237.43
Global p-value =							0.000*	0.000

# Expected genotype frequency values estimated from same generation

* Values out of HWE

^ Unable to reject null hypothesis of allele frequency changes due to genetic drift

The overall genotype frequency between all generations and replicates were not in Hardy-Weinberg equilibrium indicating that they were changing across generations (KR Fisher’s combined probability, χ^2^ = 143, p < 0.0001; CKR Fisher’s combined probability, χ^2^ = 126, p < 0.0001) (Tables 2 and 3). Based on the genetic drift model, the cumulative allele-frequency change was not due to genetic drift (KR Fisher’s combined probability, χ^2^ = 198, p < 0.0001; and CKR Fisher’s combined probability, χ^2^ = 237, p < 0.0001) (Tables 2 and 3). This indicates that the observed decrease in the *kdr* allele was due to selection (i.e. fitness cost).

### Fitness component studies

**Fig 3:**
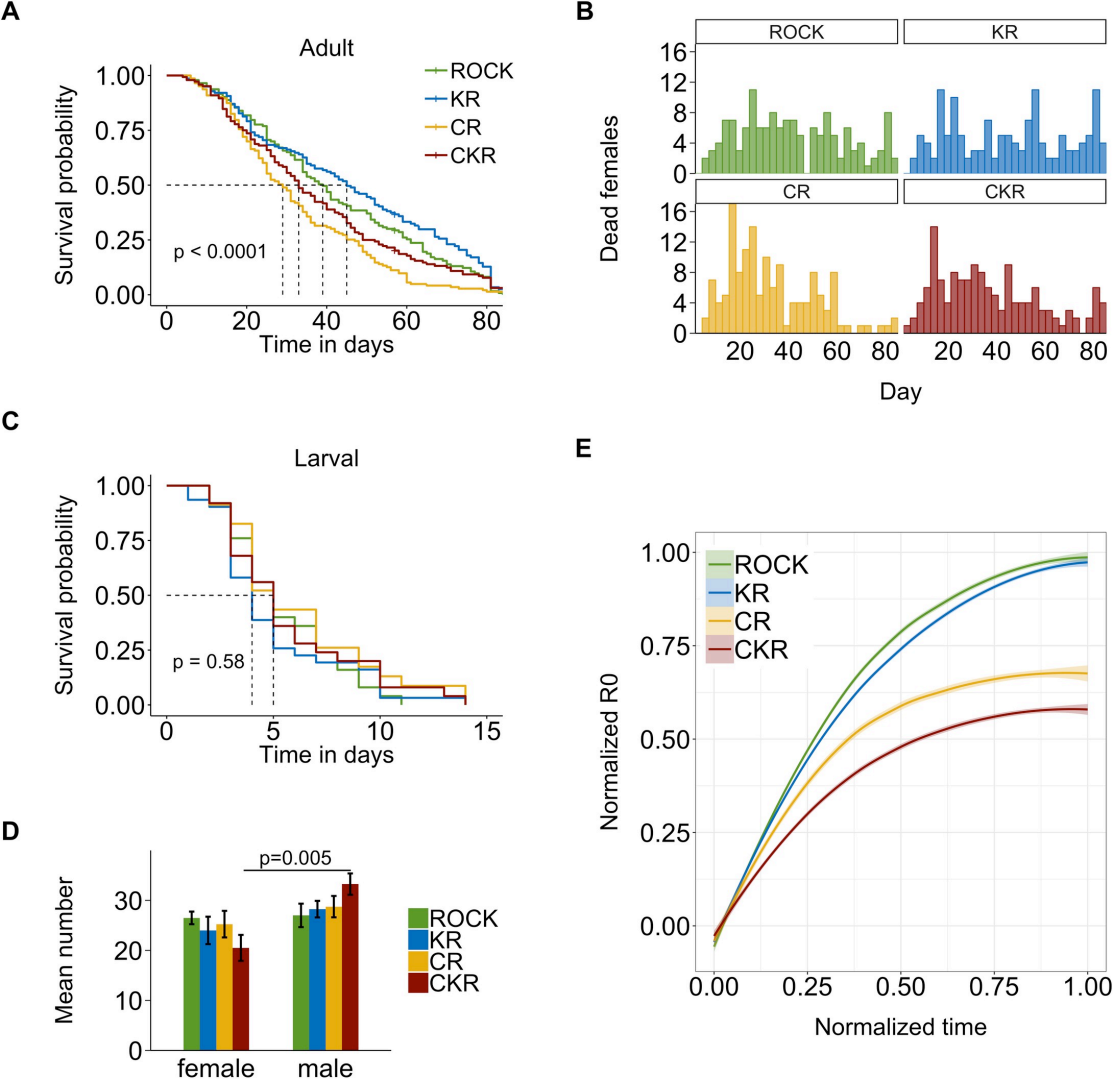
Comparison of life table components in four congenic strains of *A*. *aegypti*. (A) Kaplan-Meyer survival plot of females. (B) Average number of dead females per day for each strain. (C) Kaplan-Meyer survival curve of larvae. (D) Mean number of females and males emerged from an equal number of larvae (sex ratio). Bars represent the standard error of the means. (E) Mean R_0_ value across three replicates normalized by the highest R_0_ and time of each replicate.

The fitness costs of the different fitness components varied by strain (Table 4). Relative to the susceptible ROCK strain, KR had an approximately 4 h faster time to pupation (6.7 days relative to 6.9 days), but the KR adults did not emerge faster than ROCK or the other resistant strains (Table 4). No other components were significantly different between KR and ROCK. CR had a nine day reduction in adult survival time, but females emerged about 6 h faster than ROCK (9.2 relative to 9.4 days) (Table 4 and Fig 3A). The mortality patterns show that for CR most females (52%) die within the first 30 days, while the mortality patterns for the other strains were more uniform (ROCK = 35%, KR = 34%, and CKR = 44%) across time (Fig 3B). CKR had an approximately 1.6% reduced body size and reduced female to male ratio relative to all other strains (Table 4 and Fig 3D). Egg viability, fecundity, larval survival, and male developmental time were not different from ROCK for any of the resistant strains (Table 4 and Fig 3C). When comparing between resistant strains, KR had a longer survival time than both CR and CKR and a higher egg viability than CR (Table 4 and Fig 3A). Overall, the strains with CYP-mediated resistance had a fitness cost based on some components, but not all, and the KR strain had no fitness costs for any of the specific components measured in the adult or larval stage.

**Table 4:** Fitness components in congenic strains of *A*. *aegypti*. Values represent the mean ± SE except for sex ratio (percent female to male).

Fitness component	ROCK	KR	CR	CKR
Adult survival (days)	41.7 ± 6.0bc	45.1 ± 6.6c	32.7 ±5.0a	37.4 ± 6.0ab
Fecundity at week 1 (# of eggs)	18.0 ± 2.6a	18.0 ± 2.2a	17.2 ± 2.0a	18.2 ± 1.9a
Female body size (mm)	3.15 ± 0.02b	3.14 ± 0.02b	3.15 ± 0.02b	3.10 ± 0.02a
Sex ratio (F:M)	50:50	46:54	47:53	38:62*
Egg viability	0.75 ± 0.04ab	0.84 ± 0.02b	0.72 ± 0.03a	0.81 ± 0.03ab
Egg to pupa (days)	6.9 ± 0.1b	6.7 ± 0.0a	6.9 ± 0.0ab	6.8 ± 0.0ab
Egg to female (days)	9.4 ± 0.1b	9.3 ± 0.1ab	9.2 ± 0.1a	9.4 ± 0.1ab
Egg to male (days)	9.0 ± 0.1a	9.0 ± 0.1a	8.9 ± 0.1a	9.0 ± 0.1a
Fitness Cost Ratio	0.00 ± 0.00c	0.01 ± 0.01c	0.32 ± 0.01b	0.41 ± 0.01a
R_0_	304 ± 26b	302 ± 29b	207 ± 16a	179 ± 17a
r	0.80 ± 0.01ab	0.83 ± 0.01b	0.79 ± 0.01ab	0.75 ± 0.02a
T	7.14 ± 0.14a	6.87 ± 0.20a	6.77 ± 0.20a	6.97 ± 0.30a

Same letters within rows are not significantly different at the 0.05 level of significance. Asterisk (*) for sex ratio indicates significant difference between female to male ratio for that strain

The net reproductive rate (R_0_), intrinsic rate of increase (r), and generation time (T) were calculated based on the survival, fecundity, sex ratio, and egg viability components from the life table experiments. Combined, these values inform us about the overall reproduction and growth of the population of each strain. KR had R_0_, r, and T values that were not different from ROCK, but an R_0_ value (302 ± 47) that was different from CR and CKR (207 ± 47 and 179 ± 47 respectively) (Table 4 and Fig 3E). There was no difference in R_0_ between CR and CKR. R_0_ values varied by about 2-fold between replicates, but the trend between strains remained the same (S3 Fig). KR had a higher r value than CKR, however all r values were above 0 indicating the populations are increasing (Table 4). There was no significant difference in T between strains. Overall, the R_0_ values, but not r or T, indicate a strong fitness cost for the CYP-mediated resistance mechanism in these strains.

**Fig 4:**
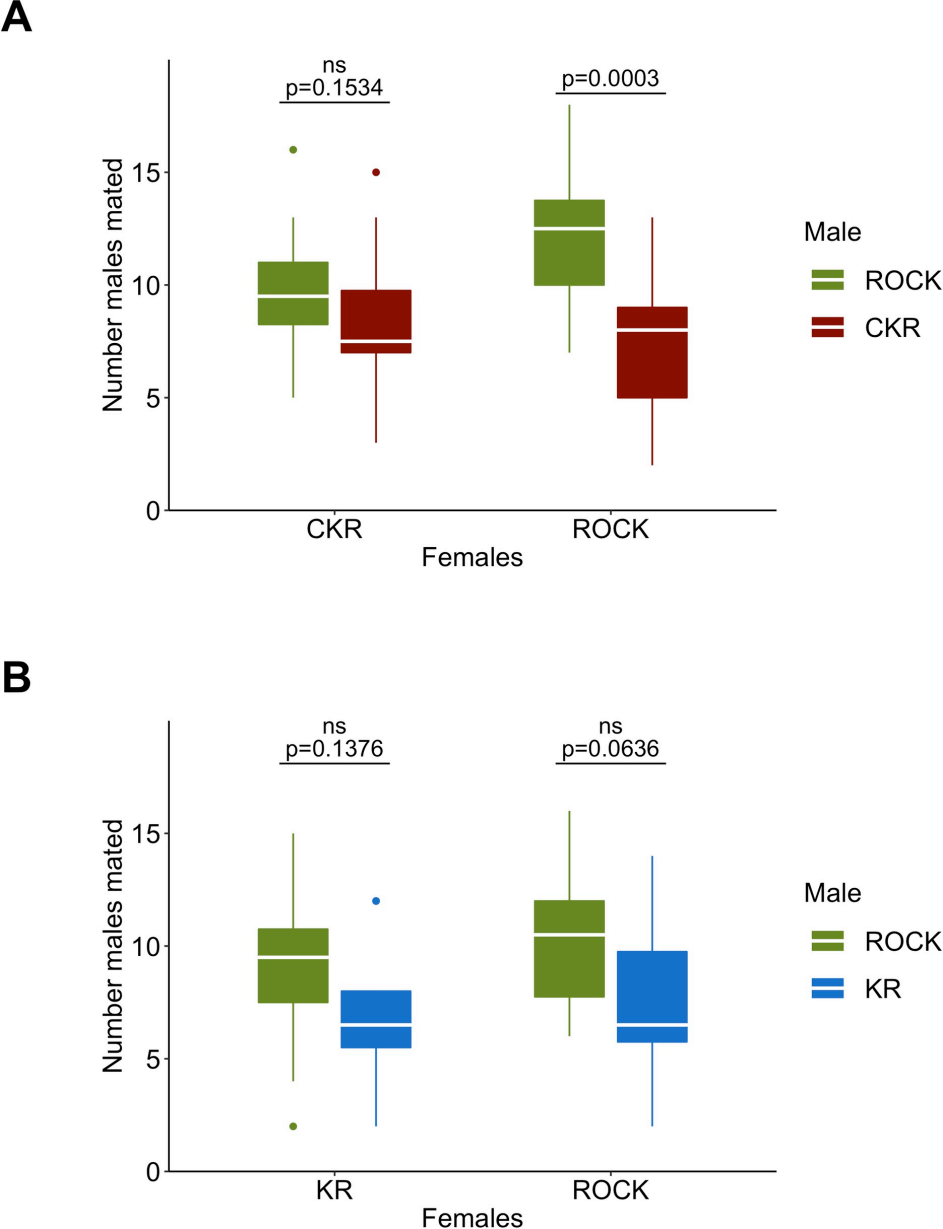
**Mating competition comparison between ROCK and either CKR (A) or KR (B) strains of *A*. *aegypti*.** Box plot of the number of males that mated with either ROCK or a resistant (CKR or KR) females. The white line in the middle represents the median, the lower and upper hinges correspond to the first and third quartiles (the 25th and 75th percentiles), the whiskers represent the minimum and maximum values no further than 1.5 times the distance between the first and third quartiles, and the points are the outliers (data beyond the end of the whiskers). ns = not statistically significant.

In the mating competition experiment between CKR and ROCK, ROCK females had a strong preference (p = 0.0003) for mating with ROCK males whereas CKR females had no preference (p = 0.145) for either ROCK or CKR males (Fig 4A). We obtained genotype results of offspring from 254 CKR females and 274 ROCK females in total with an average of about 19 females per replicate. On average, 8.2 (±1.7) CKR females mated with CKR males (45%) and 9.9 (±1.5) females mated with ROCK males (55%). An average of 7.4 (±1.6) ROCK females mated with CKR males (38%) and 12.1 (±1.6) mated with ROCK males (62%). In the mating competition experiment between KR and ROCK, neither ROCK (p = 0.064) nor KR (p = 0.137) females had a preference for mating with either ROCK or KR males (Fig 4B). For the KR versus ROCK competition, we obtained genotype results of offspring from 191 KR females and 214 ROCK females in total with an average of about 17 females per replicate. These results suggest that susceptible males are more successful at mating with susceptible females than resistant males homozygous for both *kdr* + CYP-mediated resistance, but that *kdr* alone does not have a statistically significant contribution to this effect.

**Fig 5:**
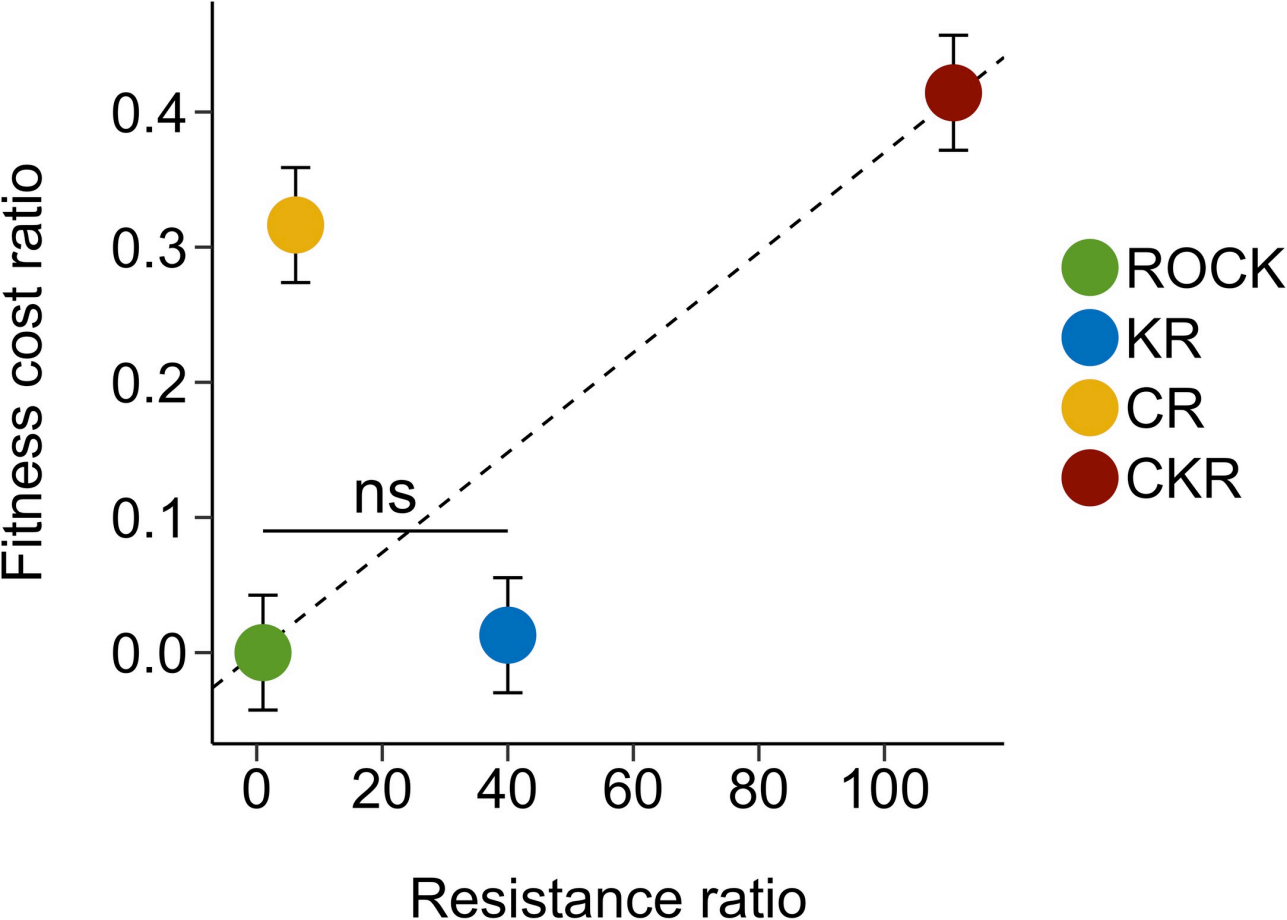
Relationship between fitness cost ratio and resistance ratio. Dashed line represents a linear relationship between fitness cost ratio and resistance ratio. Fitness ratio values are significantly different (p < 0.05) except for where labeled as “ns” (not significantly different). Bars represent the standard error of the means. Resistance ratios are from previous publications [50,51]).

To compare the fitness costs of the resistance mechanisms alone and in combination with the levels of permethrin resistance they provided, we calculated fitness cost ratios for each strain using the final R_0_ values averaged across three replicates. The fitness cost ratios were highest for the strains containing CYP-mediated resistance indicating higher fitness costs for those strains (Table 4). CKR had a significantly higher fitness cost ratio (0.41) relative to the other strains, followed by CR (0.32). The fitness cost ratios of KR and ROCK were not significantly different. When plotted against the permethrin resistance ratio levels of each strain [50,51], CKR has both the highest resistance level and the highest fitness cost (Fig 5). CR had the second highest fitness cost, but the lowest resistance level, whereas KR had no fitness cost and a moderate resistance level. The interaction between the fitness cost ratios of CR and KR was greater-than additive (p = 0.0501).

## Discussion

We found that *kdr* and CYP-mediated resistance impacts fitness costs, but they are manifested differently. Strains with CYP-mediated resistance (CR and CKR) have significantly reduced net reproductive rate (R_0_) relative to the susceptible (ROCK) and the strain containing only *kdr* (KR), indicating a higher overall fitness cost to CYP-mediated resistance than *kdr* using this metric. The fitness cost ratios (measured based on life table components) suggests that the two resistance mechanisms have a greater-than additive fitness cost effect and that there is no correlation between the fitness cost ratios and resistance levels. The *kdr* allele has a fitness cost when measured in the allele competition experiments, but not in the life table experiments. The fitness costs of CYP-mediated resistance were seen in the life table studies. This highlights the importance of different types of fitness studies to capture true fitness costs. The fitness costs of *kdr* seen in the allele-competition studies may be due to reduced fitness under a low relative humidity and high density (and thus high competition) environment. Overall, we might expect the cost of *kdr* to manifest in fitness assays where a slightly impaired nervous system could affect behavior, locomotion, etc., and we might expect the cost of CYP-mediated resistance to manifest in assays were the diversion of resources to maintain elevated CYPs is impactful, such as digestion or immunity. Consistent with these predictions, the only experiment in which we could compare and contrast the combined and individual fitness costs of *kdr* and CYP-mediated, was the life table studies where the combination of mechanisms did not radically change any of the components except sex ratio and body size. This contrasts with the greater than additive insecticide resistance the two mechanisms provide [50].

Results from fitness cost studies of pyrethroid resistance in *A*. *aegypti* are variable across the limited literature that compares related strains with known resistance mechanisms. Alvarez-Gonzalez et al. [30] found reduced egg viability, adult longevity, survival, r_m_, R_0_, and generation time, and no difference in fecundity, developmental time or sex ratio in their resistant *A*. *aegypti* strain containing CYP-mediated resistance plus a 0.5 frequency of the V1016I+F1534C *Vssc* allele and 0.5 frequency of the F1534C allele. Nearly opposite results, with reduced fecundity and developmental time, but no difference in longevity, egg viability, larval survival, and mating competition were reported for the resistant Rock-kdr strain containing *Vssc* mutations V1016I+F1534C and a slightly elevated CYP-activity [3]. Both of these strains had the same *Vssc* mutations (although in Alvarez-Gonzalez et al. the V1016I is not homozygous), but they differed in their genetic background and CYP-mediated resistance. These studies also did not check the full cDNA sequence of *Vssc* for other mutations (e.g. V410L and G923V), and this could have influenced the results. Our strains have a similar genetic background as Rock-kdr [3], but yielded very different results. Our KR strain, should be genetically similar to the Rock-kdr strain [3], except for having different *Vssc* loci (with different kdr mutations) showed no fitness costs from the life table studies. None of our strains had a reduced fecundity or differences in their developmental time. CKR’s high fitness cost ratio (i.e. low R_0_), was mostly attributed to its male skewed sex ratio that was not measured in Rock-kdr, and unlike Rock-kdr, CKR males had a lower mating success than ROCK males. A recent study by Rigby et. al (2020), found a cost in pre-adult development, female survival, and body size in an *A*. *aegypti* strain (R-BC) containing the S989P+V1016G mutations and some metabolic resistance [63]. The R-BC strain has the same *kdr* mutation as our strains, but a different genetic background and possibly different metabolic resistance. Similar to our strains, R-BC had no cost to fecundity, however, neither KR nor CKR, had a reduced survival time, body size or developmental time despite having the same *kdr* mutation. Most other resistance fitness studies done on *A*. *aegypti* have compared unrelated strains or have unknown or unclear resistance mechanisms and are therefore not useful in extrapolating a relationship between a specific resistance mechanism and its fitness costs. It is unclear if the differences between our studies and those with Rock-kdr [3] are due to the different *Vssc* mutations present, the elevated CYP-activity in Rock-kdr or something else, and whether the differences between CKR/KR and R-BC [63] are due to the different genetic background or the metabolic resistance. Based on the four studies that have now been done on different mutations and fitness costs in *A*. *aegypti*, it appears possible that the different *kdr* alleles could have significantly different fitness costs and that the costs are background dependent.

Few studies have looked at the fitness costs of CYP-mediated pyrethroid resistance without *kdr* in mosquitoes. To our knowledge, none have done so using related strains of *A*. *aegypti* prior to this study and only one has done it in *Culex* [42]. The permethrin resistant *Cx*. *pipiens quinquefasciatus* strain (IsoP450) contained only CYP-mediated resistance, and had reduced body size and nutritional content in teneral, two- and four-day old females, but no difference in survival, and developmental time [42]. Contrary to IsoP450, however, CR had a significantly reduced survival and no difference in body size. Interestingly, the developmental time, which was unchanged for IsoP450, was actually faster for CR compared to ROCK. CKR had a smaller body size and no significant difference in survival and developmental time, similar to IsoP450. CYP-mediated resistance in IsoP450 is likely due to a single CYP, *CYP9M10*, whereas in our strains, CYP-mediated resistance is due to the overexpression of several *CYPs* [20]. The fitness cost results from these two studies suggest that costs due to CYP-mediated resistance is different depending on the CYPs involved.

Previous reversion studies have found a decline in the *kdr* allele frequency over time [3,22], consistent with our results. In one allele competition study using the same Rock-kdr strain of *A*. *aegypti* described above, the *kdr* allele (V1016I+F1534C) decreased from 50% to an average of 21.7% and from 75% to an average of 20% [3]. Another reversion study, using a resistant field strain of *A*. *aegypti* reared with and without exposure to insecticide, found that the proportion of knockdown individuals at a diagnostic concentration changed from 0.14 to 0.70 over 10 generations in unselected tents [22]. They also found that the susceptible haplotype frequency (F1534+V1016) increased from 0.07 to 0.12, one resistant haplotype 1534C decreased from 0.34 to 0.14, whereas the other resistant haplotypes (1016I and 1016I+1534C) did not change (other *Vssc* mutations were not examined) [22]. These two studies support our conclusion that the different *kdr* alleles and combinations of different alleles have different fitness costs.

Although not always detected in fitness experiments, fitness costs associated with the *kdr* mutations in the absence of insecticides still exist because mutations can cause minor variations in the gating mechanisms of VSSC thus modifying the excitability of neurons and in doing so, their physiological functioning [64]. VSSCs are involved in the transmission of electrical signals in neurons through the propagation of action potentials allowing them to mediate many physiological functions in mosquitoes. Mutations in VSSC can alter its stability and conformation in molecular dynamic simulations [65] and in mammals, *Vssc* mutations have been associated with several diseases and disorders [66,67]. In insects, the biophysical functions of VSSC and the effects of mutations on these are far less understood. However, several *Xenopus* oocyte studies have demonstrated an effect of *Vssc* mutations on channel gating (see review [64]). The physiological reason for the *kdr* fitness costs we observed for both the KR and CKR strains in the allele-competition study, but not in the specific fitness component experiments, is unclear. CKR males had a clear mating competition cost when attempting to mate with the susceptible ROCK females, but had no problem competing with ROCK males when mating with fellow CKR females. This suggests that the CKR or ROCK females are making different mate choices and that ROCK females can somehow detect a deficiency in CKR (but not in KR) males that CKR females cannot. Alternatively, CKR males might be failing at mating cues, potentially due to differences in cuticular hydrocarbons [68], pheromone profiles [69], or an inability to harmonically converge [70] with ROCK females. It is worth noting that KR males also performed more poorly in the mating competition experiment compared to ROCK, even though the results were not statistically significant, indicating that *kdr* may have a fitness cost, albeit marginal, in mating competition. Further studies to determine the cause of the decline in *kdr* frequency would be interesting.

Fitness costs due to CYP-mediated resistance could be a result of a mutation in a transcription factor that targets multiple genes [71], changes in regulation of one gene that can have pleiotropic effects on other traits [72–74], or by CYP overexpression diverting resources from other fitness-enhancing traits [75]. CYPs are a vital biochemical system that metabolizes xenobiotics such as pesticides, drugs and plant toxins, and regulates the titers of endogenous compounds such as hormones and fatty acids [76], including synthesis and degradation of ecdysteroids and juvenile hormones that are essential for insect growth, development, and reproduction [77]. CYPs can also metabolize sex pheromones [78–80], are important for insect olfaction [81,82], and may be involved in circadian rhythm [83]. The effects of xenobiotic induced changes in CYP expression have been implicated with fitness cost by interfering with hormonal regulated networks [84]. In the CR and CKR strains, CYP-mediated resistance is primarily conferred by the *trans* upregulation of four CYPs [20]. In this study, fitness costs for strains having CYP-mediated resistance manifested as reduced survival time in CR, a male-biased sex ratio and smaller female body size of CKR and a reduction in R_0_ for both strains. We did not find a significant cost to reproduction (number of eggs and egg viability) between the strains having CYP-mediated resistance and ROCK. The physiological mechanisms responsible for these fitness costs remains uncertain. Identification of the mutation that gives rise to the CYP-mediated resistance would greatly facilitate future studies on this important resistance mechanism.

Many challenges and limitations exist in conducting fitness studies, which results in a gap in the literature on the fitness effects of insecticide resistance. These challenges are creation of congenic strains, the diverse and non-overlapping methods to assess fitness, and the need to know the causative mutations for resistance in the congenic strains. It is laborious and time consuming to identify resistance mechanisms [85] and to construct congenic strains. A major limitation to fitness studies is the nearly infinite number of components and environments (including field versus lab) that can be measured and how to interpret biological relevance of the different components and conditions measured. As can be seen from the results we present herein, the fitness costs of different resistance alleles manifested under some conditions, but not all. It would be beneficial to conduct field studies on the fitness costs of resistance in *A*. *aegypti*. However, optimally this would require knowing the different resistance alleles (at each locus) so that individual genotypes can be determined with high precision. We are close to being able to achieve this for the mutations in *Vssc*, as many resistance alleles are now known [86,87]. However, without knowing the allele responsible for the CYP-mediated resistance this trait cannot be accurately detected in individual mosquitoes. Our results indicate that in the presence of both *kdr* and CYP-mediated resistance at least one fitness cost is manifested (distorted sex ratio). Thus, the results from studies looking at changes in the different *Vssc* alleles over time in field populations can be significantly influenced by other resistance alleles in the population. Despite these challenges and limitations, fitness studies are still important for both our understanding of the evolution of resistance mechanisms, and whether the selective disadvantages are large enough to be useful in practical situations, such as resistance management or for new control strategies (e.g. gene editing). Lab studies using congenic strains, such as the one presented here, are important to gain preliminary understanding of how fitness costs are manifested and the potential pleiotropic effects of insecticide resistance mutations.

## Supporting information

S1 Table Genotype data. ASPCR results.
(XLSX)

S1 Fig Reproductive output study design: Summary of the life table and development experiments and how they are used to calculate R0. Survival refers to the proportion of females that survived each day. Fecundity refers to the total number of eggs laid each day (for all females in a group/strain). Egg viability refers to the proportion of eggs that hatched. Sex ratio refers to the percentage of larvae that emerged as female or male. These experiments were done for all fours strains and repeated three times.
(TIF)

S2 Fig Wing measurement image for determining body size. (TIF)

S3 Fig R0 values for each replicate across the duration of the experiment. (TIF)
