# Supplementary figures and images for: Fitness costs of individual and combined pyrethroid resistance mechanisms, kdr and CYP-mediated detoxification, in Aedes aegypti
Source: PLoS Negl Trop Dis. 2021 Mar 24;15(3):e0009271. doi: 10.1371/journal.pntd.0009271 (PMC7990171; doi:10.1371/journal.pntd.0009271)

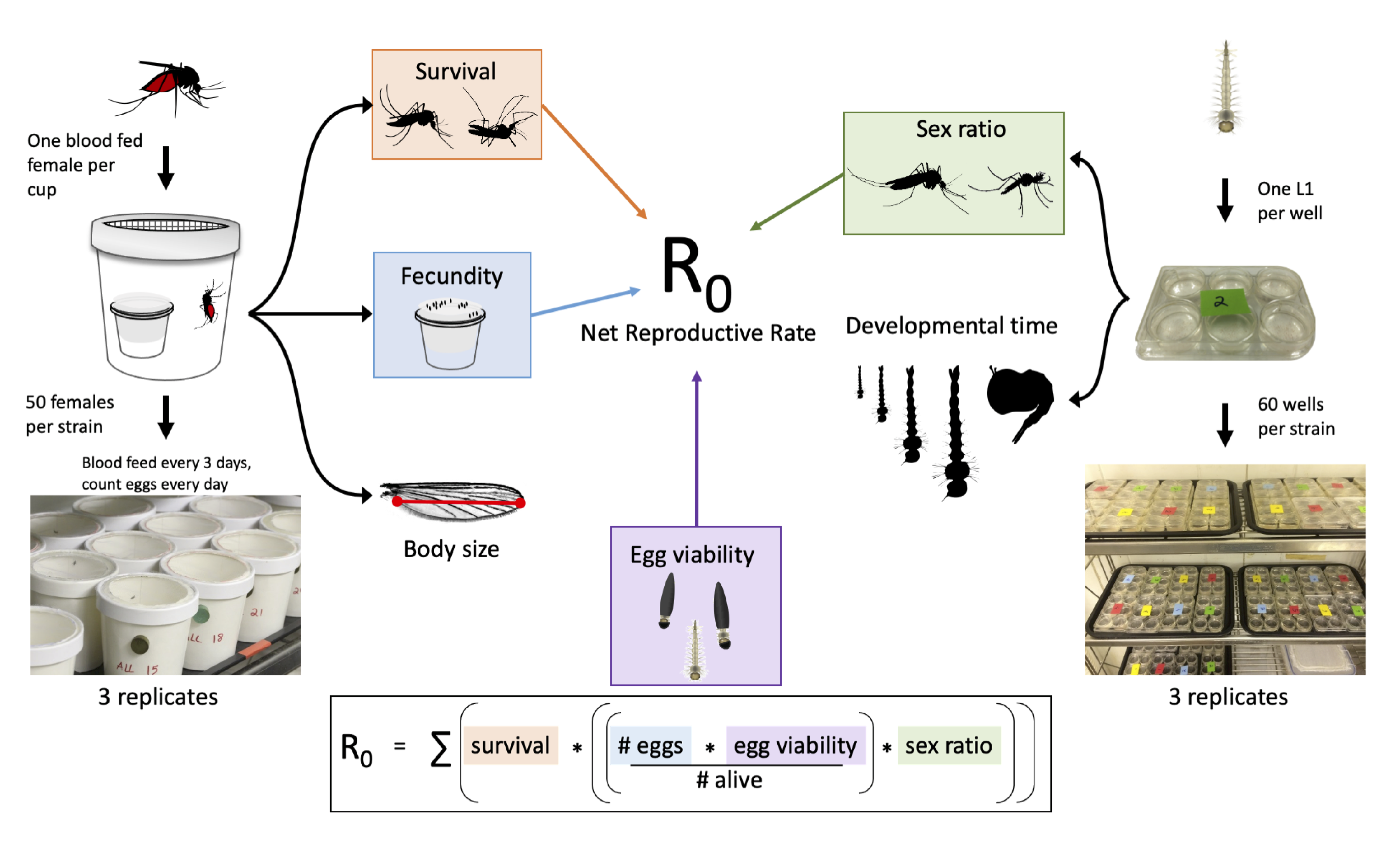

Supplement: S1 Fig — Survival refers to the proportion of females that survived each day. Fecundity refers to the total number of eggs laid each day (for all females in a group/strain). Egg viability refers to the proportion of eggs that hatched. Sex ratio refers to the percentage of larvae that emerged as female or male. These experiments were done for all fours strains and repeated three times. (TIF) [file pntd.0009271.s002.tif]

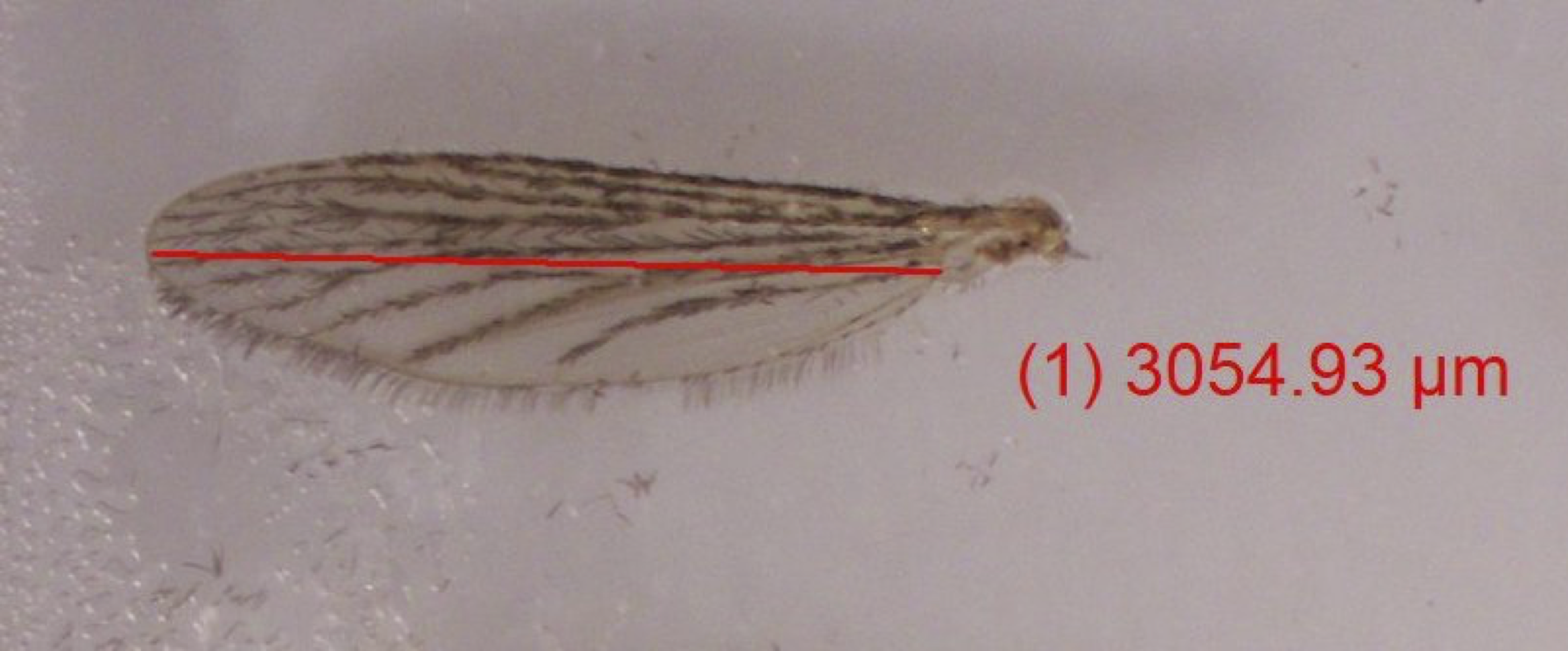

Supplement: S2 Fig — (TIF) [file pntd.0009271.s003.tif]

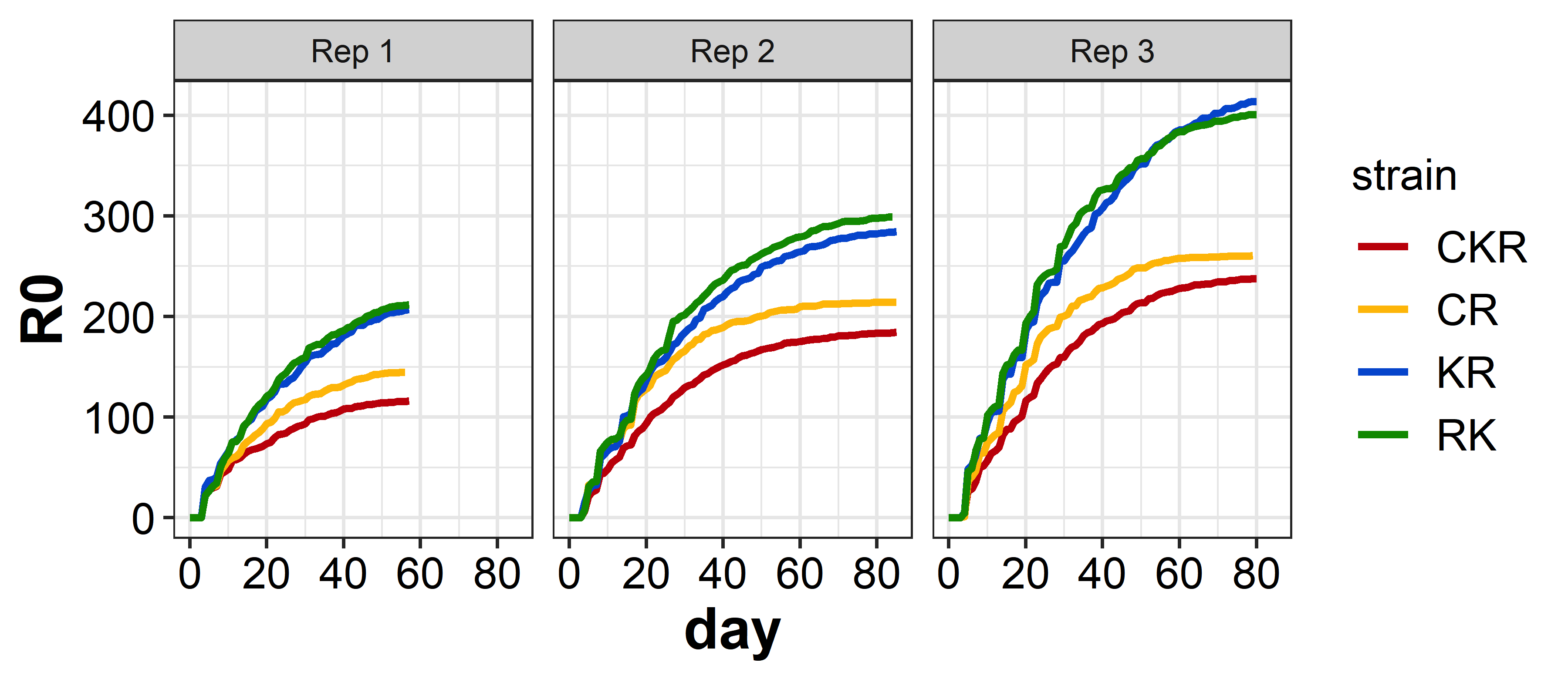

Supplement: S3 Fig — (TIF) [file pntd.0009271.s004.tif]
